# Standard setting: Comparison of two methods

**DOI:** 10.1186/1472-6920-6-46

**Published:** 2006-09-14

**Authors:** Sanju George, M Sayeed Haque, Femi Oyebode

**Affiliations:** 1 Queen Elizabeth Psychiatric Hospital, Mindelsohn Way, Edgbaston, Birmingham, UK, B15 2QZ; 2 Department of Psychiatry, University of Birmingham, Queen Elizabeth Psychiatric Hospital, Birmingham, UK, B15 2QZ

## Abstract

**Background:**

The outcome of assessments is determined by the standard-setting method used. There is a wide range of standard – setting methods and the two used most extensively in undergraduate medical education in the UK are the norm-reference and the criterion-reference methods. The aims of the study were to compare these two standard-setting methods for a multiple-choice question examination and to estimate the test-retest and inter-rater reliability of the modified Angoff method.

**Methods:**

The norm – reference method of standard -setting (mean minus 1 SD) was applied to the 'raw' scores of 78 4th-year medical students on a multiple-choice examination (MCQ). Two panels of raters also set the standard using the modified Angoff method for the same multiple-choice question paper on two occasions (6 months apart). We compared the pass/fail rates derived from the norm reference and the Angoff methods and also assessed the test-retest and inter-rater reliability of the modified Angoff method.

**Results:**

The pass rate with the norm-reference method was 85% (66/78) and that by the Angoff method was 100% (78 out of 78). The percentage agreement between Angoff method and norm-reference was 78% (95% CI 69% – 87%). The modified Angoff method had an inter-rater reliability of 0.81 – 0.82 and a test-retest reliability of 0.59–0.74.

**Conclusion:**

There were significant differences in the outcomes of these two standard-setting methods, as shown by the difference in the proportion of candidates that passed and failed the assessment. The modified Angoff method was found to have good inter-rater reliability and moderate test-retest reliability.

## Background

Kane [1] stated that the passing score is a point on the observed-score scale whereas the standard is a conceptual boundary on the true-score scale between acceptable and non-acceptable performance. Or in other words, a standard is the 'boundary between those who perform well enough and those who do not.' [2]. Standards are generally classed as absolute (criterion based) or relative (norm based) [3-5]. An absolute standard determines the pass/fail outcome by how well a candidate performs and he/she is usually judged against an arbitrarily set external standard. Hence it is independent of the performance of the group. A relative standard on the other hand, compares how well the examinee has performed compared to others who took the test and hence the outcome (pass/fail) is dependent on the performance of the group.

The outcome of assessments is determined by the standard-setting method used. Cusimano [6] defined standard – setting as "the process of deciding what is good enough". There is a wide range of standard -setting methods but the most popular ones in undergraduate medical education in the UK are the norm – reference methods and the criterion reference methods. The most widely used and researched criterion reference method of standard setting is the Angoff method [3,7]. In the Angoff method [8], a panel of judges examines each multiple-choice item or item on a checklist (for OSCEs) and estimates the probability that the "minimally competent" or "borderline" candidate would answer the item correctly. Then the scores are discussed in the group and consensus is reached if possible. This stage is avoided in the modified approach. Each judge's estimate scores on all items are added up and averaged and the test standard is the average of these means for all the judges. Each standard- setting method has its advantages and disadvantages, and there is no gold standard. The norm reference methods are easy to use and understand, can easily be explained to trainees and variations in test difficulty are automatically corrected for as the pass mark is influenced by the performance of the examinee cohort [9,10]. The drawbacks of these methods are that some examinees will always fail irrespective of their performance, students can deliberately influence the pass score and that the pass score is not known in advance [9]. On the other hand, the main advantages of the Angoff method of standard – setting are that it is widely used in a range of licensing and certifying examinations, and that it is rather well supported by research evidence [2]. However, it is not without its pitfalls. It can be very labour intensive and time consuming [3,11]. Research has also shown that judges often find it difficult to accurately conceptualise a borderline candidate [3,12-14] and often judges themselves do not feel confident of their estimates of examinee performance. Lastly, it is debatable whether Angoff method can be learnt effectively in a few hours (or in 1 training session). Studies have found [3] that judges with previous experience of Angoff method show more consensus in marking and estimating pass scores.

It is important to have an understanding of how arbitrary the judgements involved in decision-making of standard – setting can be. Glass [15] viewed all standard-setting methods that involved judges making arbitrary decisions as fundamentally flawed. Others however, argued that although all standard-setting methods require human judgement, they can be made by careful deliberation and hence be fair and reasonable. Norcini stated that although all standards are judgemental, the credibility of each standard varies depending on who sets the standards and the methods they use [16,17]. It has been found that different methods of setting standards result in different standards (see discussion) and hence it is argued that the validity of a test is determined as much by the method used to set the standard, as by the test content itself. Downing et al [18] argued that all standards are ultimately policy decisions and that 'there is no gold standard for a passing score.' What is key is the process of setting the standard. The 4 key principles that underpin the process of standard – setting are that it is systematic, reproducible, absolute and unbiased.

In this study we focussed on comparing two standard-setting methods (norm – reference and Angoff methods) and evaluating the reliability of one such method, namely the modified Angoff method. To the best of our knowledge, no previous study has explored the impact of various standard-setting methods on the outcome for candidates in a multiple-choice test in undergraduate medical education in the United Kingdom. Neither has the subject of inter-rater and test-retest reliability of the modified Angoff method been systematically studied. We set out to address these two research questions.

## Methods

This study was conducted in two phases to answer the two distinct research questions: Do different standard – setting methods result in different standards? And what is the test-retest and inter-rater reliability of the modified Angoff method?

### Phase I

The 'raw' scores of 78 4^th ^-year medical students at the Birmingham medical school on a multiple choice question (MCQ) paper in their psychiatry module were ascertained. The MCQ paper had 50, 1 in 5 best answer type items. The questions covered topics prescribed in the curriculum and were from psychopharmacology, diagnosis and classification of psychiatric disorders, management of psychiatric conditions and so on. Note that these were the real scores of real candidates on an actual MCQ paper.

The standards were set using two different standard-setting methods: the norm-reference method and the modified Angoff method. The proportion of candidates who passed or failed the test as determined by each standard-setting method was used to compare the methods.

In the norm-reference method the standard was determined by plotting the raw scores on a graph, then avoiding the 'tails' to exclude outliers and thereafter calculating an adjusted mean. The standard was arbitrarily set as the adjusted mean minus 1.0 Standard Deviation. Although arbitrary, there is some consensus among educationalists to use mean minus 1.0 SD as the pass/fail cut-off score (see discussion).

In the modified Angoff method, a panel of seven judges participated in the standard-setting exercise. They included four specialist registrars in general psychiatry, one consultant in old age psychiatry and two senior lecturers in psychiatry at the university of Birmingham. All seven were experienced in teaching medical students, were familiar with the undergraduate medical curriculum, and included a good mix of race, gender and seniority. An experienced senior examiner (F.O) introduced the Anghoff procedure and led the discussion. A consensus on the definition of a minimally acceptable, that is borderline candidate, was reached. Bearing that definition in mind, each rater judged each MCQ item and the probability that a borderline candidate would answer the item correctly. As we used the modified Angoff method, we did not have a group discussion and consensus was not established for each item. All ratings were collected and the mean of each rater's total judgment scores on all 50 items was calculated. This mean score indicates, in the rater's judgement, the score that a minimally competent candidate would obtain.

### Phase II

In this phase, the test-retest and the inter-rater reliability (separately for phases I and Phase II) of the modified Angoff method of standard -setting were estimated. This was conducted 6 months after phase I. We used the same MCQ paper that was used in phase I of the study. A panel of 5 raters was selected, 3 of who had participated in phase I (two specialist registrars and one senior lecturer in psychiatry) and two had not (two specialist registrars in psychiatry). Again, all raters were very familiar with the undergraduate medical curriculum. The same format was used: introduction to the topic by F.O, group discussion on the concept of a "borderline" candidate and individual ratings of each MCQ.

### Statistical analyses

#### Calculation of pass score

The data were analysed using SPSS version 10.0 by a statistician (M.S.H). To calculate the pass score using the norm reference method, we used a 'trimmed mean' – i.e. we plotted the raw scores on a graph and then excluded the extreme 5% to avoid the influence of outliers [19]. The pass score was set at mean minus 1 SD. The modified Angoff method of standard setting used in our study is described in detail in the methods section.

#### Comparison of the two methods and calculation of inter-rater and test-retest reliability

Comparison of the Angoff and norm reference methods was done by looking at their percentage agreement, which was determined by calculating the percentage of cases (students) that gets the same result (pass or fail) by the 2 different methods (see Table I for results). 95% confidence interval of this agreement was also estimated. The inter-rater reliability of the Angoff method was checked by using Intra Class Correlation Coefficient (ICC) [20] employing the average method of reliability and two-way random effects model. We used this model because our judges were a random sample (of all possible judges) and the questions were also a random selection. Finally, the test-retest reliability of the Angoff method was established by calculating Pearson's correlation coefficient between phase I and phase II scores for the same three raters.

### Ethical approval

Ethical approval was not required for this study.

## Results

### Results by the two methods and their comparison

The pass rate with the norm-reference method i.e. mean minus 1.0 SD was 85% (66/78) and that by the modified Angoff method was 100% (78 out of 78). As noted earlier the choice of mean minus 1.0 SD as the pass/fail cut -off score was entirely arbitrary (although this is common practice among educationalists). The two methods of standard – setting, i.e. norm – reference (mean minus 1.0 SD) and modified Angoff method, were compared by looking at the percentage agreement between them. The percentage agreement between the Angoff and the norm – reference method was 78% (95% Confidence Interval = 69% – 87%).

### Angoff method

#### Inter-rater reliability

In Phase I, seven clinicians were involved in creating the pass scores for the modified Angoff method. Average scores of the seven clinicians were found to be 58.17; therefore, the pass mark was set at 58. The Intraclass Correlation Coefficient (ICC) measured the inter-rater reliability of the seven clinicians. The ICC calculated as the average measure of reliability and by using two-way random effects model was 0.82 (95% CI 0.73 – 0.89). This indicates that there was very good inter-rater reliability. The same inter-rater reliability for the five clinicians in Phase II was found to be 0.81 (95% CI 0.71 – 0.88), also suggesting a high inter-rater reliability.

#### Test-Retest reliability

Out of the seven clinicians who participated in Phase I of the study, only three clinicians participated in Phase II. The test-retest reliability was established by calculating Pearson's Correlation coefficients between the Phase I and Phase II scores for these raters. The results are presented in Table 1 below.

The correlation coefficients demonstrate moderately good test-retest reliability.

## Discussion

This study had two important limitations. First, the number of raters who participated in the Angoff method of standard setting was small: seven in phase I and five in phase II. There is no clear consensus among researchers on the most appropriate number of judges. Norcini and Shea [16] commented that around 5 to 10 judges would be acceptable, whereas Hurtz and Hertz [21] recommend a number of 10 to 15, and Zieky and Livingston [22] suggested between 5 and 30 judges. However, Cizek [23] was more realistic in stating that one should use as many judges as resources permit. There is also some evidence that increasing the number of judges improves the reproducibility of the passing score [9]. Hence, although the number of judges used in our study might be acceptable, we recognise that a larger panel size would have probably yielded more valid findings. The second limitation of our study was the choice of mean minus 1 SD as the pass/fail cut-off score in the norm – reference method of standard setting – this was entirely arbitrary. We opted for this cut-off as it has been previously used in other studies by educationalists. Verhoeven et al [10] in a study that assessed the credibility of the Angoff method (by comparing it with a normative method) used mean minus 1 SD as the cut-off. A similar method was also adopted in another study that looked at the reliability and credibility of Angoff method in undergraduate medical examination using recent graduates as judges [9].

It is also worth emphasizing some of the strengths of our study. Correctly defining and accurately operationalizing the concept of a 'borderline candidate' is crucial to the Angoff method. Boursicot and Roberts [3] referred to the idea of a borderline or minimally competent candidate as a 'nebulous concept' and research has shown that often judges find it difficult to accurately define and understand a hypothetical borderline student [18,24]. We used the following definition -'the borderline examinee is one who has an exactly 50:50 probability of passing or failing the test. The borderline examinee is the marginal student-one who on some days might just barely pass your assessment but on other days might fail.' [18] Downing et al [18] noted that asking the judges to describe such a student from their experience and then facilitating a discussion within the group often improved the judges' understanding of this concept – this is the process we adopted in our study. In our study we also avoided a group discussion among judges and no 'reality check' (giving information on actual performance data) was provided -again similar to previous research [25]. This we acknowledge was a rather 'purist' view – not presenting judges with actual performance data. Although, providing a 'reality check' has been found to improve the credibility of the Angoff method, it does not always improve the reliability. Another strength of our study was the careful attention paid to selecting judges for the Angoff method, as 'the passing score established is only as credible as the judges.' [16]. Downing et al [18] highlights the key aspects to consider in selecting judges as their content expertise, familiarity with the examinee cohort and a good balance in gender, ethnicity, seniority and sub specialisation [18,26] – our panel fulfilled all these requirements.

Our study found that in determining the outcome of a multiple choice question paper for a cohort of medical students there is limited agreement between the modified Angoff method and the norm-reference method. The pass rate with the norm – reference method was 85% and that by the Angoff method was 100%, and the percentage agreement between the two was only 78% (Confidence Interval = 69% – 87%). Or stated simply, these two different standard setting methods yielded different standards. This finding is similar to that reported in previous studies [9,18,27-30]. Verhoven et al [10] compared the pass/fail rates (on an Individual Statement Questions Examination used in undergraduate medical assessment) derived from the modified Angoff method and the norm – reference method (mean minus 1 SD) and found them to be significantly different – failure rates of 56.5% and 10.1% respectively. Similarly, studies have also applied different standard setting methods to OSCEs in undergraduate medical examinations and have found the standards set to be very different [24,28]. Although it is now fairly well established that different standard setting methods result in different outcomes or passing scores, they can be made credible, defensible and acceptable by ensuring the credibility of judges and using a systematic approach to collect their judgements [18].

Our study also found that the inter-rater reliability (0.81 – 0.82) and the test-retest reliability (0.59–0.74) of the standard determined by the modified Angoff method were very good and moderately good respectively. We recognise that the numbers of raters in this study is small particularly with regard to our calculation of test-retest reliability, and this caveat needs to be borne in mind while interpreting our findings. In trying to compare our study findings on the reliability of the modified Angoff method, note that 'there is no consensus on the definition of the modified Angoff method.' [31]. We took it to mean that no correction was applied for guessing [10], there was no group discussion [25] and no 'reality check' was done [25] – all having been adopted in previous studies. Verhoeven et al [9] assessed the reliability of the Angoff method in a progress test, using the generalizability theory and found the reliability to be 0.90 (Crobach's alpha) for the total PT. We found the test-retest reliability to be 0.59 to 0.74 – a contrastingly low value as compared to previous studies. Wayne et al [24] noted very good inter-rater reliability (ICC = 0.88 – 0.98) and test -retest reliability (0.95 – 0.98). for the Angoff method. A reliability coefficient of 0.8 or more is considered satisfactory in high stakes examination [11]. However, the Angoff method when applied to OSCEs has also been shown to have poor inter-rater reliability. Bourscot and Roberts [3] compared the passing scores in an OSCE (same stations) across 5 UK medical schools and the inter-rater reliabilities ranged from 0.36 to 0.49. Reliability estimates of the Angoff method have also used different panel composition on the same test – Verhoven et al [10] compared item writers to recent graduates and found that recent graduates showed more agreement and produced more reliable estimates. In order to confirm that our findings of high inter-rater reliability and moderate test-retest reliability are robust, future studies using a larger sample size of panel members need to be done.

## Conclusion

Conventional standard setting methods such as the norm – reference method are arbitrary, whereas the modified Angoff method of standard setting is more objective and has good inter-rater reliability and moderate test-retest reliability. Although the proportion of candidates that passed or failed varied considerably with the method of standard setting used, there was some agreement between the norm – reference and the Angoff methods of standard setting. The Angoff method has self-evident face validity as it replaces grossly arbitrary methods with a reasoned, standardised method that is open to inquiry. Nonetheless there is need to further investigate the statistical characteristics of the modified Angoff method in order to establish its limits and strengths, and its use in undergraduate medical examinations.

## Competing interests

The author(s) declare that they have no competing interests.

## Authors' contributions

FO conceived the idea for the study. SG and FO organised and co-ordinated the project and MSH carried out the statistical analysis. All authors were involved in the interpretation of data and writing the paper.

## Pre-publication history

The pre-publication history for this paper can be accessed here:



## Figures and Tables

**Table 1:** Test-retest reliability of 3 raters by Pearson's correlation coefficient

	**Correlation Coefficient**	**2-tail P-value**
**Rater 1**	0.66	<0.001
**Rater 2**	0.74	<0.001
**Rater 3**	0.59	<0.001
